# Triple combination of HAIC-FO plus tyrosine kinase inhibitors and immune checkpoint inhibitors for advanced hepatocellular carcinoma: A systematic review and meta-analysis

**DOI:** 10.1371/journal.pone.0290644

**Published:** 2023-10-16

**Authors:** Zhongbao Tan, Jian Zhang, Lan Xu, Huanjing Wang, Xuequn Mao, Rong Zou, Qingqing Wang, Zhuang Han, Zhenhai Di, Daguang Wu

**Affiliations:** 1 Department of Interventional Radiology, The Affiliated Hospital of Jiangsu University, Jiangsu University, Zhenjiang, Jiangsu, China; 2 Department of Oncology, Funing County People’s Hospital, Yancheng, Jiangsu Province, China; 3 Department of ICU, The Affiliated Hospital of Jiangsu University, Jiangsu University, Zhenjiang, Jiangsu, China; Al-Azhar University, EGYPT

## Abstract

**Background:**

The triple combination of hepatic arterial infusion chemotherapy (HAIC) with fluorouracil, leucovorin, and oxaliplatin (FOLFOX) plus tyrosine kinase inhibitor (TKI) and immune checkpoint inhibitors (ICIs) is expected to have a synergistic anticancer effect in HCC. We conducted this meta-analysis to evaluate the efficacy and safety of the triple combination treatment in advanced HCC patients.

**Methods:**

PubMed, Embase, Cochrane Library, Web of Science databases were systematically searched for relevant studies from the inception of each database to May 10, 2023. All articles focusing the triple combination treatment of HAIC-FO plus TKI and ICIs for advanced HCC were eligible. The meta-analysis was conducted following the PRISMA guidelines. The risk of bias was assessed using the Joanna Briggs Institute (JBI) for case series and Newcastle-Ottawa Scale (NOS) for cohort studies. The primary outcomes were overall survival (OS), progression-free survival (PFS), objective response rate (ORR) and disease control rate (DCR). The secondary results were adverse events. Further meta-analysis of control studies demonstrated the superiority of the triple combination modality to TKI plus ICIs, and TKI alone.

**Results:**

Nine articles (four cohort studies and five one-arm studies) involving 777 advanced HCC patients were included in this meta-analysis. In terms of survival analysis, the pooled median PFS was 11 months (95% CI: 10.1–12.0 months) with low heterogeneity (I^2^ = 0%, *p* = 0.97). With regard to tumor response, the pooled ORR and DCR was 61.6% (I^2^=0%, *p* = 0.71) and 87.9% (I^2^ = 13%, *p* = 0.33) with low heterogeneity, respectively. As compared with TKIs plus ICIs, and TKIs alone, the triple combination thrapy was associated with improved median OS (HR=0.51, 95%CI 0.41-0.62) with low heterogeneity across studies (I^2^ = 0%, *p* = 0.47), median PFS (HR=0.51, 95%CI 0.41-0.64) with low heterogeneity across studies (I^2^ = 0%, *p* = 0.41), ORR (RR = 0.56, 95% CI: 0.42–0.74) with high heterogeneity across studies (I^2^ = 69%, *p* = 0.02), and DCR (RR = 0.38, 95%CI 0.27–0.54) with low heterogeneity across studies (I^2^ = 14%, *p* = 0.32). The most common 3/4 AEs were elevated ALT and AST, thrombocytopenia, hypertension, nausea and vomiting in this meta-analysis.

**Conclusions:**

The triple combination therapy of HAIC-FO plus TKI and ICIs showed promising efficacy and safety in patients with advanced HCC.

**Registration:**

The protocol was registered with PROSPERO (ID:CRD42023424281).

## Introduction

Hepatocellular carcinoma (HCC) is one of the most common malignant tumors and the third leading cause of cancer-related deaths worldwide [1]. Multi-target tyrosine kinase inhibitor, such as sorafenib, lenvatinib, donafenib, regorafenib and cabozantinib, have found widespread clinical applications for HCC. In addition to TKI, ICIs such as programmed cell death 1 (PD-1)/ programmed cell death ligand 1 (PD-L1) inhibitor, pembrolizumab, atezolizumab, nivolumab, sintilimab, camrelizumab, tislelizumab and durvalumab, have revolutionized malignancy tumor therapy in recent years. The Barcelona Clinic of Liver Cancer (BCLC) group recommended systemic treatment with multi-target tyrosine kinase inhibitor (TKI) and immune checkpoint inhibitors (ICIs) as the standard therapies for patients with advanced HCC (BCLC C) [2]. Recently, Hepatic arterial infusion chemotherapy (HAIC) using fluorouracil, leucovorin, and oxaliplatin (FOLFOX) has been carried out as an alternative or integrative method for advanced HCC patients. HAIC has been identified as cost-effective and non-inferior to transarterial embolization (TACE) in advanced HCC, especially massive tumor and major vascular invasion [3–5]. HAIC as an alternative therapy is recommended for advanced HCC in China, Japan, Korea and other Asian countries [6–8]. A meta-analysis reported that sorafenib plus HAIC improved OS, PFS, and ORR compared with sorafenib alone in advanced HCC [9]. In a nationwide, retrospective, cohort, real-world study (CHANCE001) in china, Zhu et al. [10] demonstrated that TACE plus ICIs and molecular targeted therapies could significantly improve PFS, OS, and ORR versus TACE alone in advanced HCC. Several clinical trials [11–19] have shown that the triple combination of HAIC-FO plus TKI and ICIs could improve tumor response and survival in advanced HCC. Nevertheless, all studies were non-randomized controlled trials with small sample sizes. Therefore we conducted this meta-analysis to evaluate the efficacy and safety of the triple combination therapy of HAIC-FOLFOX (HAIC-FO) plus TKI and ICIs, hoping to provide a more effective basis for the triple combination treatment of advanced HCC.

## Material and method

This meta-analysis study was conducted according to the Preferred Reporting Items for Systematic Reviews and Meta-Analysis (PRISMA) guideline(S1 File); we have registered at the International Prospective Register of Systematic Reviews (PROSPERO, review registry ID: CRD42023424281).

### Search strategy

PubMed, Embase, Cochrane Library, Web of Science databases were systematically searched for relevant studies from the inception of each database to May 10, 2023. The following search terms were used: “primary liver cancer” or “liver tumor” or “hepatocellular carcinoma” or “HCC”, and “hepatic arterial infusion chemotherapy”or “HAIC”, and “tyrosine kinase inhibitors” or “TKI” or “sorafenib” or “sunitinib” or “linifanib” or “lenvatinib” or “donafenib” or “bevacizumab” or “targeted therapy” or “molecular targeted therapy”, and “nivolumab” or “pembrolizumab” or “atezolizumab” or “camrelizumab” or “durvalumab” or “tislelizumab” or “toripalimab”or “PD-1” or “PD-L1” or “immunotherapy” or “Immune Checkpoint Inhibitors” or “ICI”.

### Inclusion and exclusion criteria

The literature chosen for the conduction of this study should comply with the following inclusion criteria: (1) advanced hepatocellular carcinoma; (2) triple combination of HAIC, TKI, and ICIs; (3) progression-free survival (PFS), overall survival (OS), objective response rate (ORR), disease control rate (DCR), and adverse events (AEs) were reported or calculated using related data; (4) studies with original data (case series, cohort, retrospective, case–control studies, randomized controlled trials); (5)published in English.

The exclusion criteria were as follows: (1) non-clinical studies, such as guidelines, reviews, meta-analyses, case reports, letters, conference papers, comments, abstracts and animal experiments; (2)studies with data unrelated to the triple combination of HAIC, TKI, and ICIs; (3) articles lacking relevant outcome measures.

### Data extraction

After relevant articles were identified from the above databases, two independent reviewers (ZB.T, Z.H) reviewed the data separately. Once there was an argument, a third reviewer (J,Z) was invited to settle the disagreement. The following information will be extracted from the included studies: (1) primary author’s name, year of publication and country of study; (2) study design, sample size, age, sex, Child-Pugh class, ECOG-PS, BCLC stage, AFP, tumor size, extrahepatic metastasis and vascular invasion; (3) treatment strategy; (4) outcomes: OS, PFS, objective response rate (ORR), disease control rate (DCR), and AEs. Tumor responses were evaluated according to the modified Response Evaluation Criteria in Solid Tumors (mRECIST) [20]. AEs were evaluated using the National Cancer Institute Common Terminology Criteria for Adverse Events (CTCAE 5.0) [21].

### Quality assessment

Analysis and quality evaluation of the literature were conducted independently by two authors. All articles were evaluated for completeness of outcome data and selective reporting of research outcomes. Risk of bias for case series was independently assessed using the appropriate Joanna Briggs Institute (JBI) Critical Appraisal Checklists [22]. The quality assessment of cohort studies was evaluated using the Newcastle-Ottawa Scale (NOS), which contained three aspects: comparability, selection, and result evaluation [23]. Studies with a NOS score greater than or equal to 6 were considered as high-quality(S2 File). Studies with a Joanna Briggs Institute (JBI) score of 0-14 were considered as low quality and 15-20 as high quality(S4 File).

### Statistical analysis

All statistical analyses were performed using R software (version 4.2.3). Survival results, such as OS and PFS, were pooled as hazard ratio (HR) with 95% confidence interval (CI) using Forest plots. ORR and DCR were pooled as the risk ratio (RR) with 95% CI which were displayed in Forest plots. Statistical heterogeneity was evaluated using I^2^ statistics and chi-square test. A fixed effects model was adapted if the I^2^ value was less than 50%; otherwise, a random-effect model was used. Sensitivity analyses were conducted by removing each study individually when heterogeneity was observed. A p-value less than 0.05 meant statistical significance.

## Results

### Searching results

**Fig 1:**
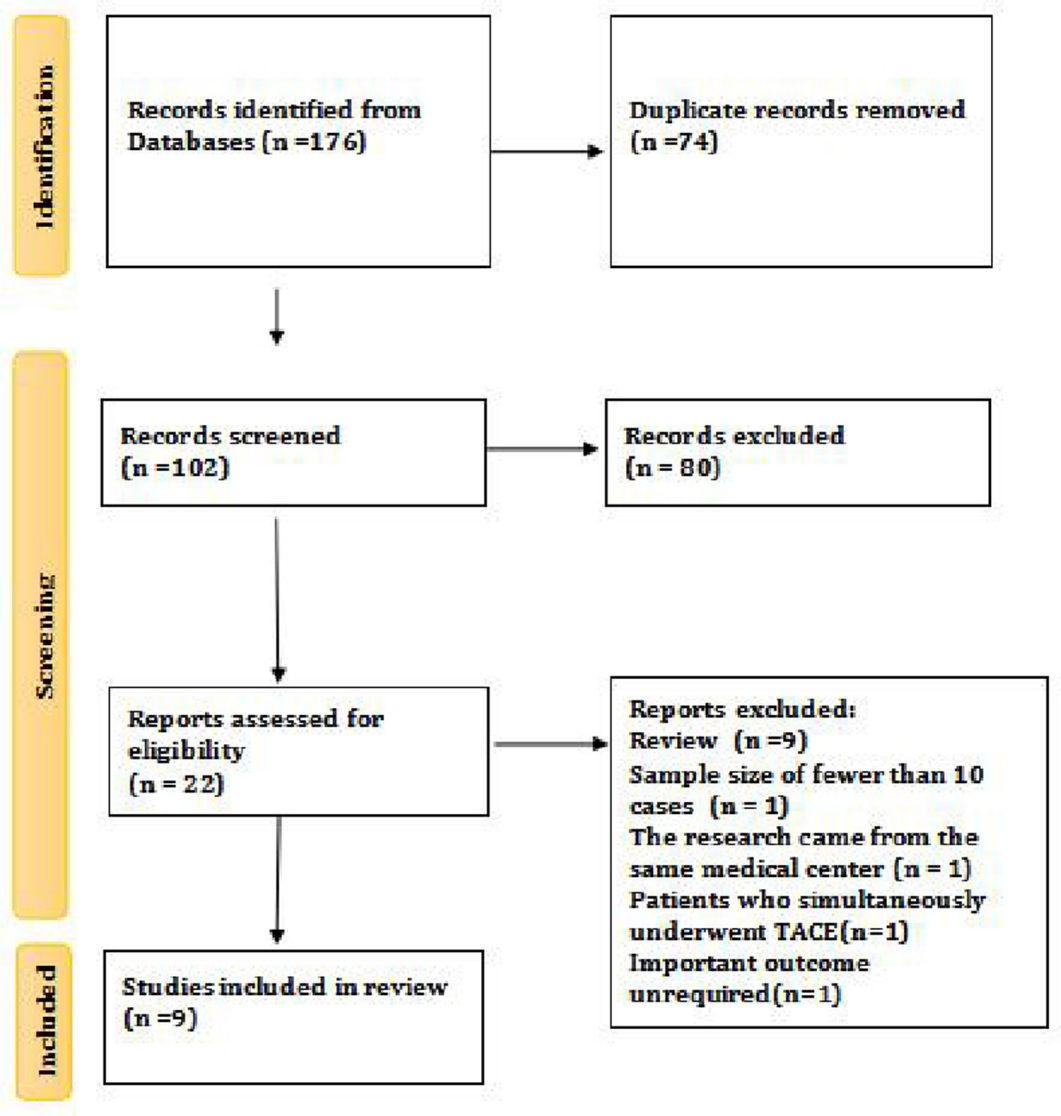
Flowchart of study inclusion.

A total of 176 potentially relevant studies were identified through the systematic literature search, including 37 in MEDLINE, 19 in Cochrane Library, 68 in Embase and 52 in Web of Science (Fig 1). 74 duplicates were excluded. After exclusion of ineligible articles and reference lists of relevant articles by screening of title and abstract, 9 studies met the inclusion criteria and were included in the final analysis. Summary characteristics of included studies are presented in Table 1.

**Table 1:** Baseline characteristics of the studies.

Authors	Country	Design	Year	Treatment	HAIC-FO	Cases(n)	Age(years)	Sex(M/F)	Child-Pugh(A/B)	PS(0/1/2)	BCLC(B/C)	AFP (≤400/ >400mg/L)	Tumor size(cm)	Extrahepatic metastasis(Y/N)	MVI(Y/N)	NOS/JB I
Chen. et al. [11]	China	RT	###	HAIC -Len-P	85mg/m^2^ oxaliplatin from hour 0 to 2 on day 1; 400 mg/ m^2^ fluorouracil bolus at hour 3 and 2400 mg/m^2^ fluorouracil over 46 h on days 1 and 2; and 400 mg/m2 leucovorin from hour 2 to 3 on day 1	84	52 (42–67)	72/12	71/13	38/46	22/62	3984.0 (82.0–49,534.0)	NA	20/64	35/49	9
				P-Len		86	53 (43–69)	71/15	75/11	35/51	21/65	4022.0 (79.0–51,462.0)	NA	24/62	41/45	
He. et al. [12]	China	RT	###	HAIC-Len-P	85mg/m^2^ oxaliplatin from hour 0 to 2 on day 1; 400 mg/ m^2^ fluorouracil bolus at hour 3 and 2400 mg/m^2^ fluorouracil over 46 h on days 1 and 2; and 400 mg/m2 leucovorin from hour 2 to 3 on day 1	71	40(≤50)31(>50)	59/12	NA	14/57	NA	26/45	26(≤10)/45(>10)	16/55	16/55	8
				Len		86	42(≤50)44(>50)	77/9	NA	22/64	NA	31/55	40(≤10)/46(>10)	25/61	24/62	
Mei. et al. [13]	China	RT	###	HAIC-Len-P	85 or 135 mg/m^2^ oxaliplatin, 400 mg/m^2^ leucovorin, and 400 mg/m^2^ fluorouracil on day 1; and 2400 mg/m^2^ fluorouracil over 46 h.	45	49.1 ± 10.6	38/7	44/1	NA	B5/C40	4106.0 (72.8,121000)	11.2 ± 3.9	15/30	9/38	6
				Len		25	50.1 ± 12.3	18/7	22/3	NA	B3/C22	767.6 (23.3, 21940.5)	10.9 ± 4.2	13/12	7/18	
Liu. et al. [14]	China	RT	2021	HAIC-TKI-P	Oxaliplatin, 60–75 mg/m^2^ HAIC for 0–4 h; [Child–Pugh A, 75 mg/m^2^; and Child–Pugh B7, 60 mg/m^2^], 5- fluorouracil, 1-1.5 g/m^2^ HAIC for 4–24 h [Child–Pugh A, 1.5 g/m^2^; and Child–Pugh B, 1 g/m^2^]) and leucovorin ([200 mg, 2 h before 5-Fu])	27	59.2 ± 1.4	26/1	22/5	18/9/0	C27	12/15	77.4 ± 0.71	8/19	7/20	18
Zhang. et al. [15]	China	RT	2021	HAIC-TKI-P	Oxaliplatin 85 mg/m^2^ 2 hours, calcium folinate 400 mg/m^2^ as a 2–3 hours and fluorouracil 400 mg/m^2^ as a bolus injection, followed by fluorouracil 1200 mg/m^2^ administered over 23 hours on day 1.	25	62(49∼78)	19/6	22/3	NA	C25	NA	NA	NA	25	18
Luo. et al. [16]	China	RT	2022	HAIC-TKI-P	HAIC with oxaliplatin, 5-fluorouracil, and leucovorin and RALOX (HAIC with raltitrexed plus oxaliplatin).	145	61(≤50)84(>50)	121/24	116/29	138/7/0	B41/C104	63/82	72(<10)/73(≥10)	33/112	70/75	17
Xin. et al. [17]	China	RT	2022	HAIC+A+T	Oxaliplatin at 85 mg/m^2^ for 2–4 h, leucovorin at 400 mg/m^2^ for 2 h, fluorouracil at 400 mg/m^2^ for 1 h, and 2400 mg/m^2^ fluorouracil over 46 h.	52	20(<55)32(≥50)	46/6	NA	49/3	NA	22/30	22(<10)/30(≥10)	26/26	15/37	18
Xu. et al. [18]	China	RT	2022	HAIC-Len-P	Oxaliplatin 85mg/m^2^ hour 0–2 on Day 1, leucovorin 200 mg/m^2^ hour 2–3 Day 1, fluorouracil 400 mg/m^2^ hour 4 on Day1 and 2400 mg/m2 fluorouracil over 46 h.	61	39(<60)22(≥60)	53/8	53/8	13/41/7	B9/C52	38/23	39(<10)/22(≥10)	44/17	15/46	18
Fu. et al. [19]	China	RT	###	HAIC-Len-P	Oxaliplatin 130mg/m^2^ hour 0–2 on Day 1, leucovorin 200 mg/m^2^ hour 2–3 Day 1, fluorouracil 400 mg/m^2^ hour 4 on Day1 and 2400 mg/m2 fluorouracil over 46 h.	45	49.1 ± 10.6	38/7	44/1	NA	5/40	4106.0 (72.8, 121000.0)	11.2 ± 3.9	15/30	9/36	8
				Len-P		25	50.1 ± 12.3	18/7	22/3	NA	3/22	767.6 (23.3, 21940.5)	10.9 ± 4.2	13/12	7/18	

RT: retrospective study; P: PD1/PDL1; Len: lenvatinib; A +T: a tezolizumab +bevacizumab; PS: Performance Status; Y:yes; N: None. NA: not available.

### Study characteristics and quality assessment

Five one-arm and four retrospective cohort studies published between 2021 and 2023 were included (777 patients). The triple combination therapy of HACI-FO plus lenvatinib and ICIs were reported in four retrospective cohort studies comparing the two combination of lenvatinib and ICIs in two studies, lenvatinib alone in two studies. The remaining five one-arm studies analyzed the efficacy and safety of the triple combination therapy. Majority of patients had a well compensated liver function (Child–Pugh A). The baseline characteristics of the studies were shown in Table 1.

### Tumor response

**Fig 2:**
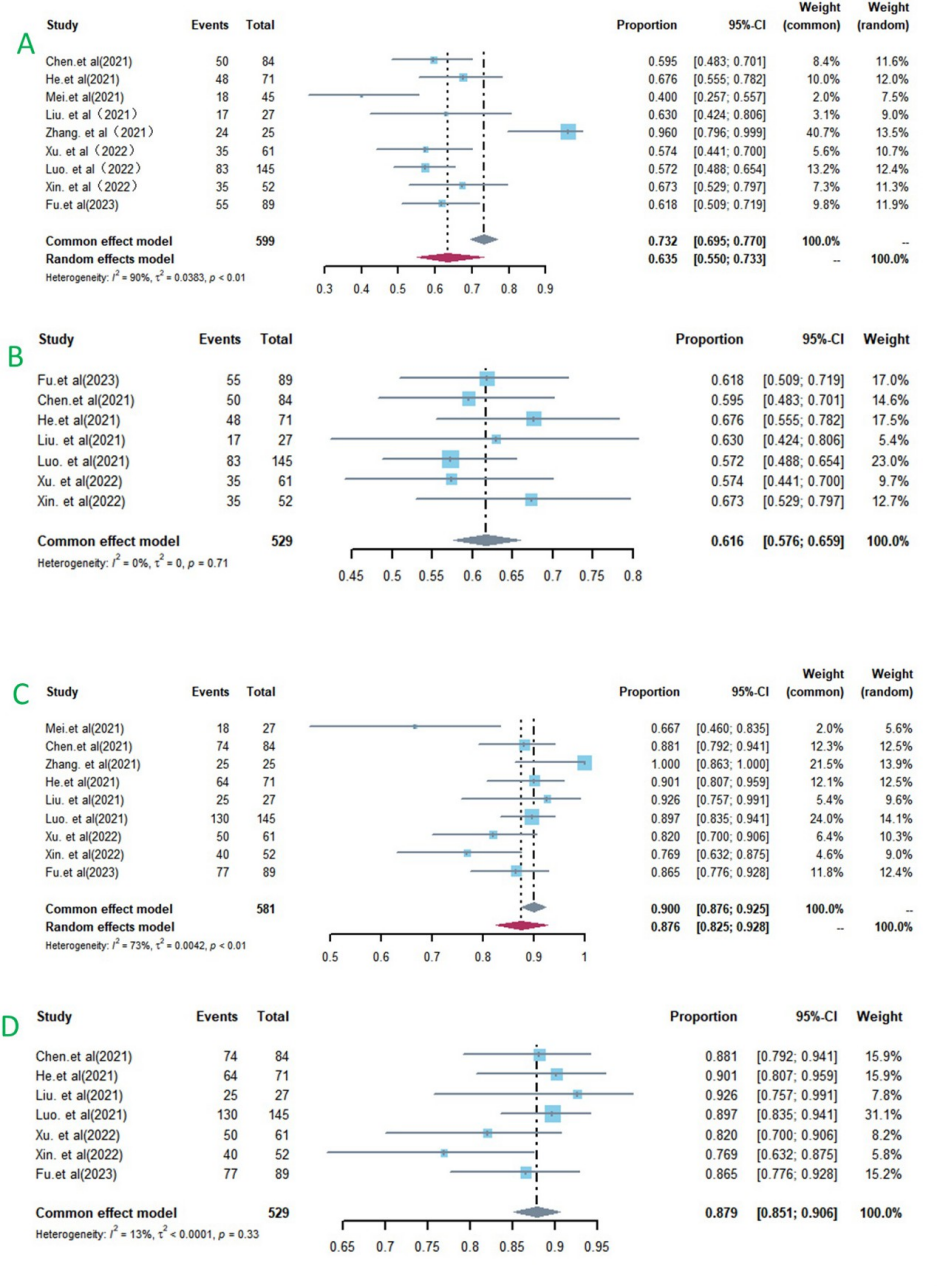
Forest plot about the pooled results of HAIC-FO plus TKI and ICIs for advanced HCC. Outcome: ORR (A,B) and DCR (C,D) in total. ORR, objective response rate; DCR, disease control rate.

The ORR across the studies varied from 40% to 96% with a significant heterogeneity (I^2^ = 90%, *p* < 0.01) (Fig 2A). The pooled DCR was 87.6% (95%CI: 82.5%–92.8%), with high heterogeneity (I^2^ = 73%, *p* < 0.01) (Fig 2C). Sensitivity analysis by excluding each study in individually suggested that Mei et al.’s and Zhang et al.’s study were the source of heterogeneity. Unable to evaluate tumor respone of ten patients in Mei et al.’s study(10/70) and high surgical conversion rate(14/25) attributed to the heterogeneity of ORR and DCR. After removing the two studies, the pooled ORR was 61.6% (95%CI: 55%-73%) with low heterogeneity(I^2^=0%, *p* = 0.71) (Fig 2B) and the pooled DCR was 87.9% (95% CI: 85.1%–90.6%) with low heterogeneity (I^2^ = 13%, *p* = 0.33)(Fig 2D).

### Progression-free survival

**Fig 3:**
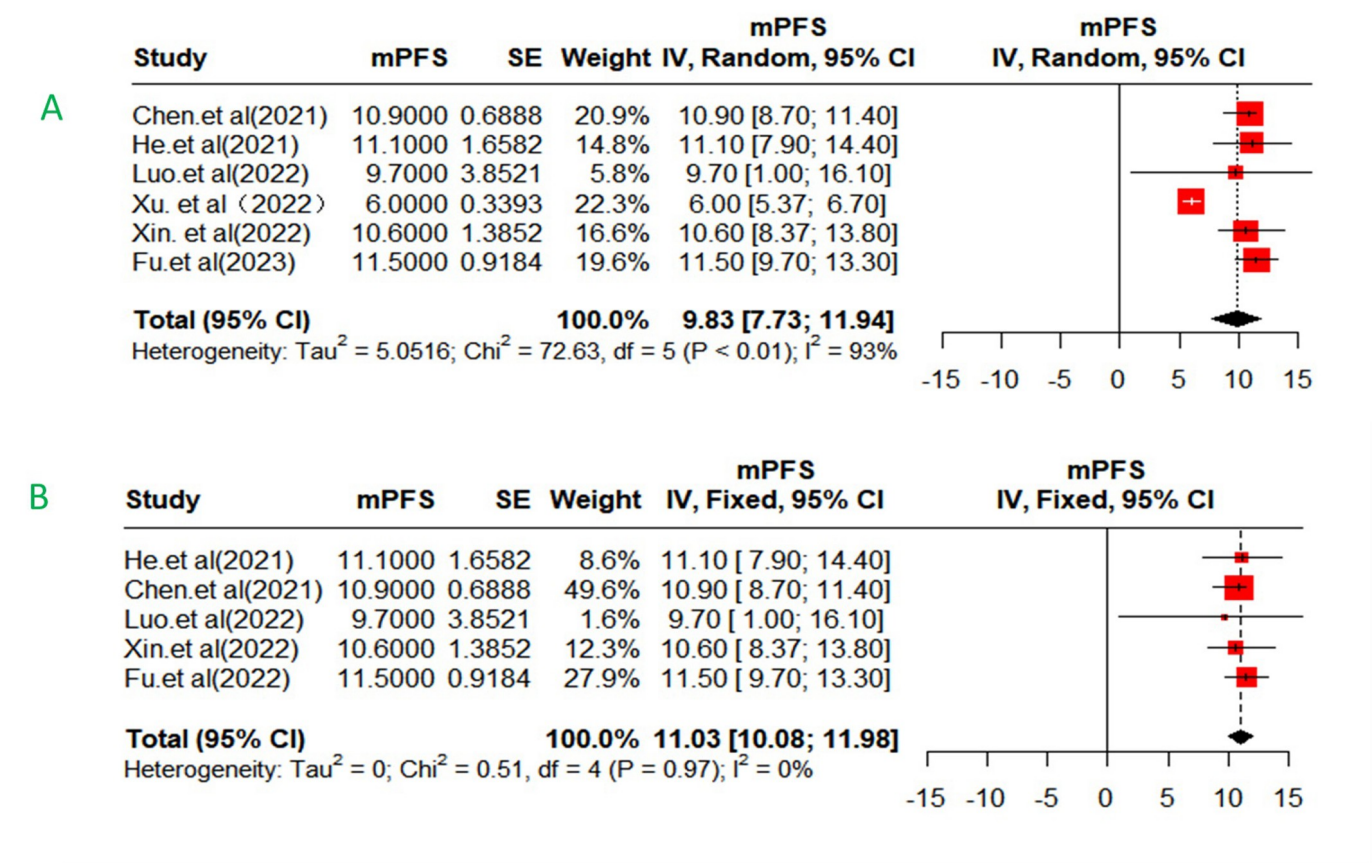
Forest plot about the pooled mPFS in total with HAIC-FO plus TKI and ICIs for advanced HCC(A,B). mPFS, median progression-free survival.

The median OS was not reached in five studies; therefore, we could not perform meta analysis of OS. The median PFS was not reached in two studies (Liu et al.’s and Zhang et al.’s). Meanwhile, in Mei et al.’s study, the median PFS was not provided. Finally, six of these studies were included in the final analysis. The pooled mPFS was 9.8 months (95%CI: 9.7-13.3months) with high heterogeneity (I^2^ = 93%, p < 0.01) (Fig 3A). Sensitivity analysis by excluding each study in individually suggested that Xu et al.’s study, which had a short follow-up, high proportion of PVTT (68.1%) and extrahepatic metastasis (68.1%), was the source of heterogeneity. After removing the study, the pooled mPFS was 11 months (95% CI: 10.1–12.0 months) with low heterogeneity (I^2^ = 0%, *p* = 0.97) for patients receiving HAIC-FO plus TKI and ICIs (Fig 3B).

## Subgroup analysis of cohort studies

### Survival of HAIC-FO plus TKI and ICIs vs. TKI with or without ICIs

**Fig 4:**
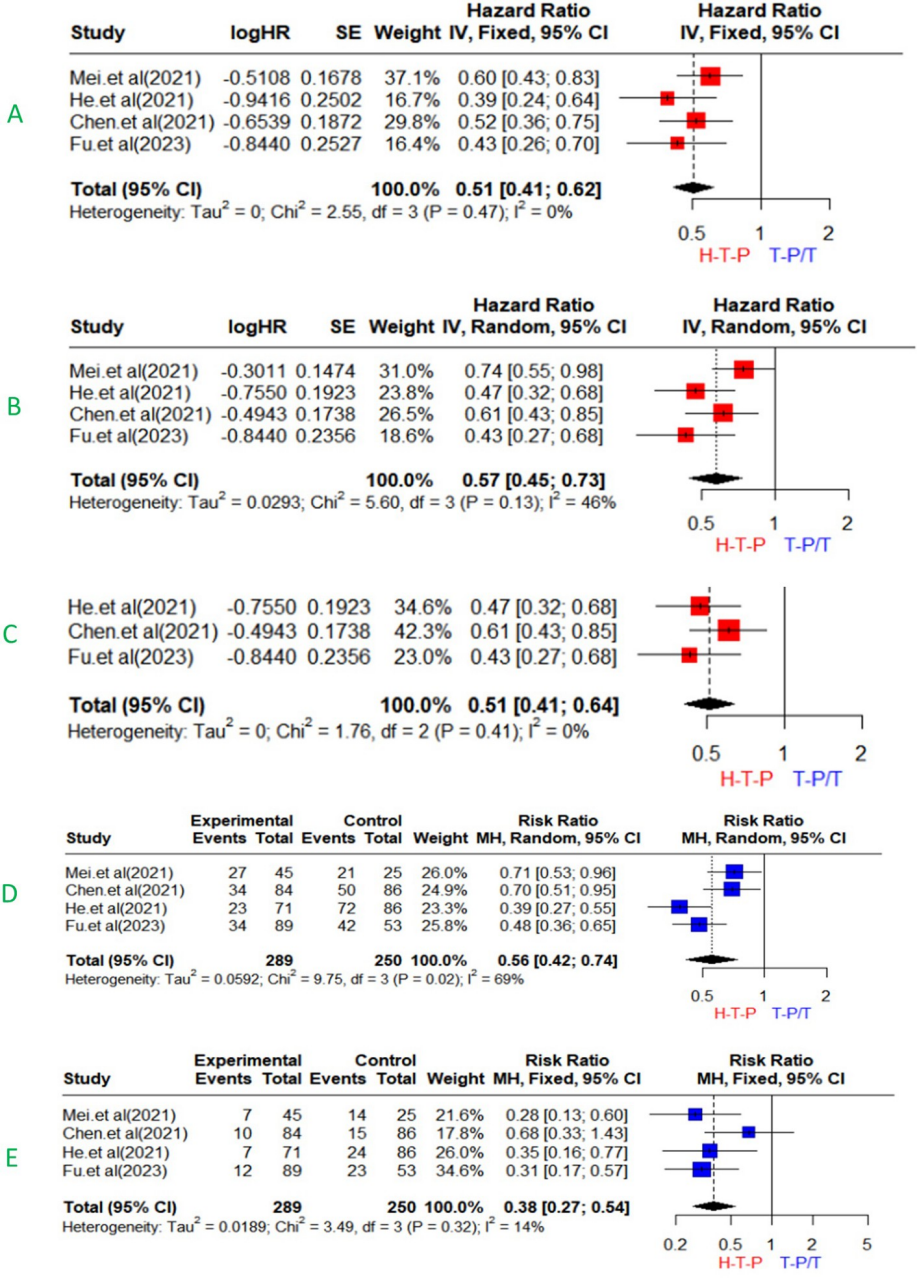
Forest plots for HAIC-FO plus TKI and ICIs for advanced HCC. Outcomes: (A): OS; (B, C): PFS; (D): objective response rate (ORR), (E): disease control rate (DCR).

Our meta-analysis demonstrated that the triple combination therapy of HAIC-FO plus TKI and ICIs was superior to TKI with or without ICIs in patients with advaned HCC (HR=0.51, 95%CI 0.41-0.62). The heterogeneity was low (I^2^ = 0%, *p* = 0.47) (Fig 4A). Regarding PFS, the results suggest that, compared to TKI with or without ICIs, the triple combination therapy could prolong mPFS (HR=0.57, 95%CI 0.45-0.73) with moderate heterogeneity (I^2^ = 46%, *p* = 0.13) (Fig 4B). Sensitivity analysis by excluding each study individually suggested that Mei et al.’s study was the source of heterogeneity. After excluding Mei et al.’s study, the final result of mPFS favored the triple combination therapy (HR=0.51, 95%CI 0.41-0.64) with low heterogeneity(I^2^ = 0%, *p* = 0.41) (Fig 4C).

### Efficacy of HAIC-FO plus TKI and ICIs vs. TKI with or without ICIs

Our meta-analysis demonstrated that, compared with TKI with or without ICIs, the triple combination therapy improved the ORR (RR = 0.56, 95% CI: 0.42–0.74) with high heterogeneity (I^2^ = 69%, *p* = 0.02), and the DCR (RR = 0.38, 95%CI 0.27–0.54) with low heterogeneity(I^2^=14%, *p* = 0.32) (Fig 4D).

### Adverse events

**Fig 5:**
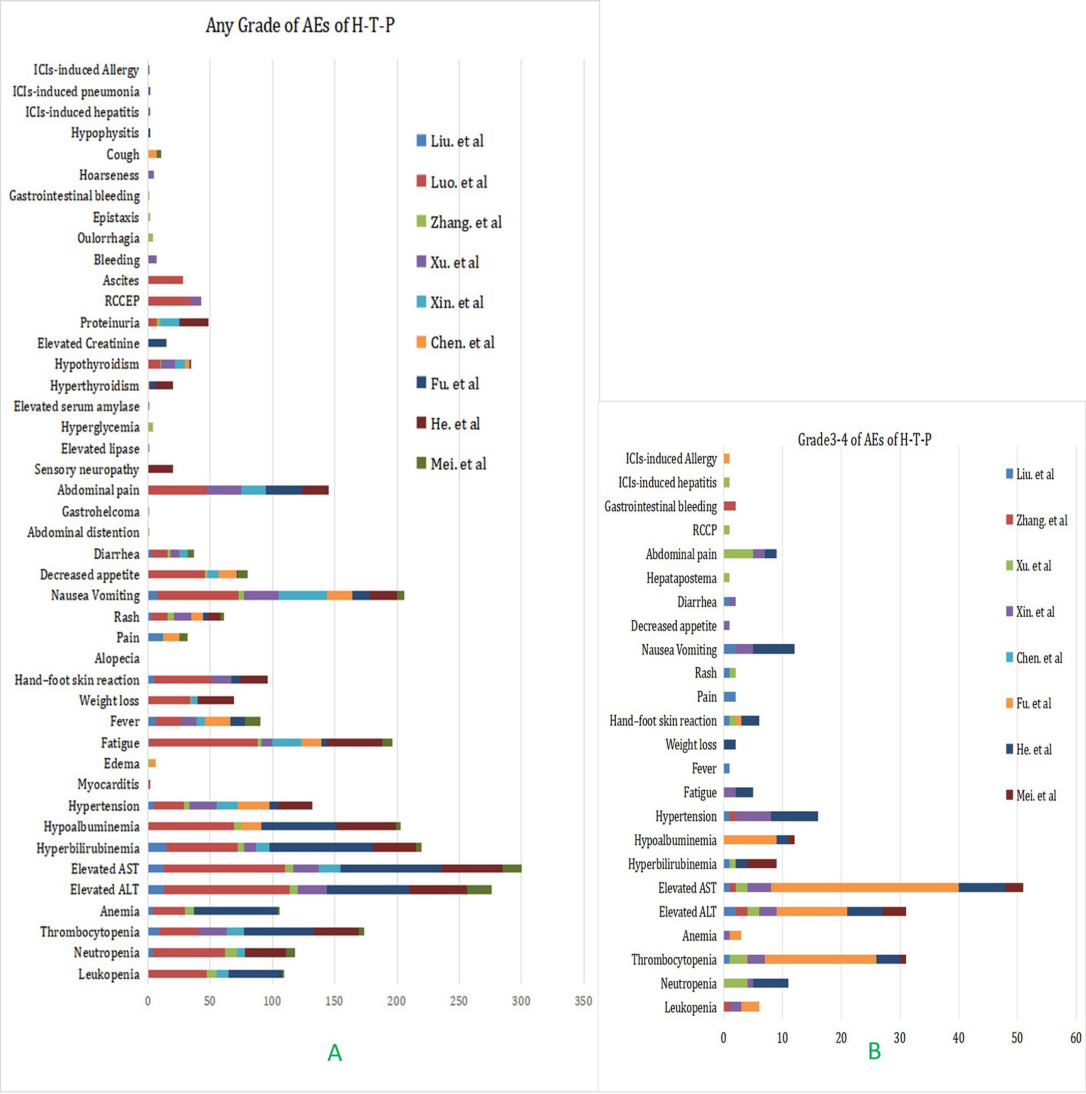
Adverse events: Any grades (A) and grade 3/4(B) in total.

A total of nine studies reported relevant data on treatment-related toxicity of the triple combination therapy (S3 File). The most common AEs were ALT/AST elevation, hyperbilirubinemia, hypoalbuminemia, nausea, vomiting, fatigue, thrombocytopenia, abdominal pain, hypertension (Fig 5A). The most common relative risk for grade >=3 treatment-related AEs were ALT/AST elevation, thrombocytopenia, hypertension, nausea and vomiting (Fig 5B).

### Publication bias

According to the Cochrane Handbook, the publication bias was not assessed as a limited number of studies included in each meta-analysis was less than 10.

## Discussion

To our knowledge, this meta-analysis is the first to evaluate the efficacy and safety of the triple combination of HAIC-FO plus TKI and ICIs for advanced HCC. This meta-analysis reported the pooled analyses of nine studies (five one-arm and four retrospective cohort studies) with 777 patients who underwent a triple combination of HAIC-FO plus TKI and ICIs for advanced HCC. In total, the pooled mPFS was 11 months (95% CI: 10.1–12.0 months) with low heterogeneity (I^2^ = 0%, *p* = 0.97). The pooled ORR was 61.6% (95%CI: 55%-73%) with low heterogeneity (I^2^=0%, *p* = 0.71) and the pooled DCR 87.9% (95%CI: 85.1%–90.6%) with low heterogeneity (I^2^=13%, *p*=0.33) after sensitivity analysis. The results of our meta-analysis demonstrate that, compared with TKI with or without ICIs, the triple combination of HAIC-FO plus TKI and ICIs improved the OS, PFS, ORR and DCR. The most common relative risk for grade >=3 treatment-related AEs were ALT/AST elevation, thrombocytopenia, hypertension, nausea and vomiting.

Currently, HAIC with chemotherapeutic agents (such as oxaliplatin, 5-fluorouracil, cisplatin, gemcitabine, floxuridine, epirubicin, individually or in combination) delivered via a catheter or pump is regarded as one of alternative treatment options for patients with advanced HCC. In Japan, HAIC is recommended for HCC patients with major portal vascular invasion and in patients with intrahepatic multinodular lesions who are ineligible for hepatectomy, RFA, TACE [7]. In a phase II trial conduced in China, treatment with HAIC-FO in patients with advanced HCC showed promising efficacy and tolerable toxicity with a median time to progression (TTP) of 6.1 months and response rate of 28.6% (RECIST v1.1) or 40.8% (mRECIST) [24]. HAIC-FO is recommended as an alternative therapy for advanced HCC in China [6]. HAIC attracted more attentions in recent years. HAIC-FO was evaluated as compared with sorafenib in 262 patients with advanced HCC in a randomized, phase III trial (FOHAIC-1). The median OS was 13.9 months for HAIC-FO and 8.2 for sorafenib (HR=0.408; 95% CI, 0.301 to 0.552; p <0.001) [25]. Systemic treatment using tyrosine kinase inhibitor and immunotherapy have demonstrated survival benefit for advanced HCC [26–29]. The triple combination therapy of HAIC-FO plus TKI and ICIs is expected to provide additional survival benefit.

In this meta-analysis, the ORR varied from 40% to 96% with a significant heterogeneity. In the Mei et al.’s study, the low ORR may caused by unevaluatable clinical data on tumor response. Using the triple combination therapy of HAIC-FO plus TKI and ICIs, the conversion rate was reported to be as high as 56% (14/25) by Zhang et al.’s study, which led to the high heterogeneity in ORR and DCR. After excluding above study, our meta-analysis showed significant effects with a pooled ORR of 61.6% (95%CI: 55%-73%). This ORR is similar to that reported with the triple combination therapy of TACE plus TKI plus ICIs for advanced HCC (60.1%) (10). In this meta-analysis, the pooled mPFS was 11 months, which is longer compared to the TACE plus TKI plus ICIs in Zhu et al.’s study (9.5 months) [10]. Our meta-analysis demonstrated that, compared with TKI plus ICI combination therapy or TKI monotherapy, the triple combination of HAIC-FO plus TKI and ICIs improved the OS and PFS in patients with advanced HCC. The triple combination therapy of HAIC-FO plus TKI and ICIs maybe a good choice for advanced HCC.

Several limitations should be considered in our study. Firstly, immunotherapy for HCC is an emerging treatment in recent years. Few studies using the triple combination therapy of HAIC-FO plus TKI and ICIs has been carried out for the treatment of advanced HCC. None RCT trial was used to evaluate the triple combination therapy of HAIC-FO plus TKI and ICIs for advanced HCC. Secondly, various kinds of TKI and ICIs agents in the different studies might have influenced the response of HCC. However, all TKI and ICIs applied in these studies are recommended for HCC either in the west or in Chinese guidelines. Thirdly, all of the included studies were conducted in China. In China, HBV infection is common in HCC patients. It remains unclear whether the differences of demographic characteristics and backgrounds in different countries will influence the effectiveness of the triple combination therapy. Fourthly, there was no consensus on the regimens of HAIC, the scheme in each study was a little different from each other. The heterogeneity might relate to the dose and duration of HAIC in different protocols of treatment in this study. However, all these studies have utilized oxaliplatin in combination with fluorouracil, and leucovorin. Finally, the baseline characteristics of each study included were not identical, which might affect the heterogeneity.

In conclusion, our meta-analysis demonstrates the efficacy and safety of the triple combination therapy of HAIC-FO plus TKI and ICIs in patients with advanced HCC, and the triple combination therapy is superior to TKI plus ICI combination therapy or TKI monotherapy. However, since there are limited clinical data, RCTs with large sample are required to confirm this conclusion. Excitingly, clinical trials of HAIC combined with targeted drugs and immune checkpoint inhibitors are ongoing (NCT05313282, NCT05582278, NCT05250843, ChiCTR2200061735, ChiCTR2100046555, etc.), and more effective results are expected to guide clinical practice.

## Supporting information

S1 Checklist PRISMA 2020 checklist. (DOCX)

S1 File (XLSX)

S2 File (XLSX)

S3 File (TIF)

S4 File (XLSX)
