# Supplementary figures and images for: Triple combination of HAIC-FO plus tyrosine kinase inhibitors and immune checkpoint inhibitors for advanced hepatocellular carcinoma: A systematic review and meta-analysis
Source: PLoS One. 2023 Oct 16;18(10):e0290644. doi: 10.1371/journal.pone.0290644 (PMC10578571; doi:10.1371/journal.pone.0290644)

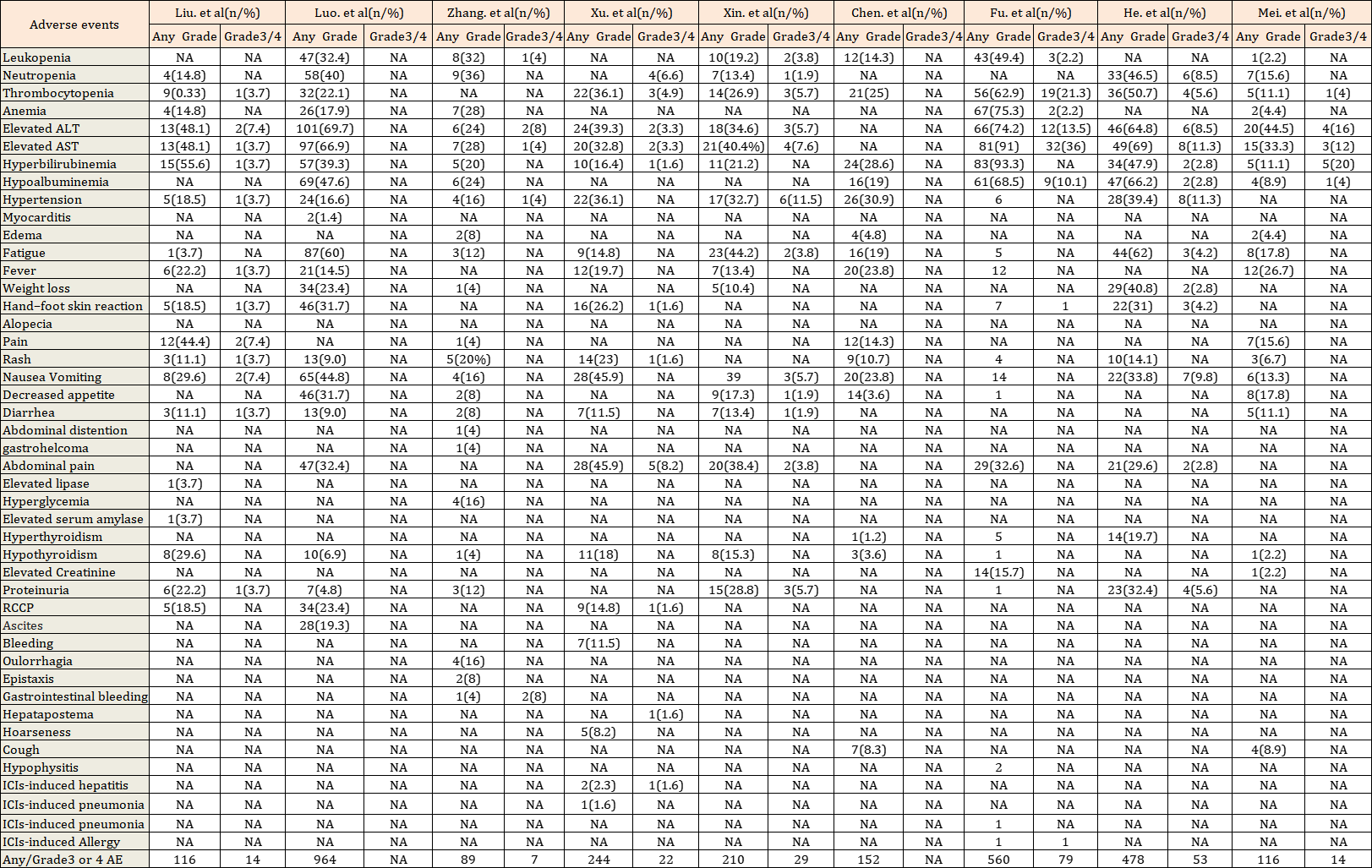

Supplement: S3 File — (TIF) [file pone.0290644.s004.tif]
